# A PDM-based bi-directional fibre-FSO integration with two RSOAs scheme

**DOI:** 10.1038/s41598-019-44904-x

**Published:** 2019-06-05

**Authors:** Wen-Shing Tsai, Hai-Han Lu, Yong-Cheng Huang, Shi-Cheng Tu, Qi-Ping Huang

**Affiliations:** 1 Department of Electrical Engineering, Ming Chi University of Technology, New Taipei City, 243 Taiwan; 2 Institute of Electro-Optical Engineering, National Taipei University of Technology, Taipei, 106 Taiwan

**Keywords:** Atmospheric optics, Fibre optics and optical communications

## Abstract

A polarization-division-multiplexing (PDM)-based bi-directional fibre-free-space optical (FSO) integration with two reflective semiconductor optical amplifiers (RSOAs) scheme to efficiently wipe off the modulated data for upstream modulation is proposed and successfully demonstrated. For downstream modulation, a high-speed 128 Gb/s vestigial sideband (VSB)-four-level pulse amplitude modulation (PAM4) fibre-FSO integration is feasibly established. The transmission capacity is increased up to four times through PDM operation and VSB-PAM4 modulation. For uplink transmission, a 10 Gb/s non-return-to-zero fibre-FSO integration with two RSOAs scheme to effectually erase the downstream modulated data is practically constructed. The upstream performance exhibits noticeable enhancement by using of two RSOAs scheme to wipe off the modulated data clearly. Such illustrated PDM-based bi-directional 128 Gb/s (downstream)/10 Gb/s (upstream) fibre-FSO integration is shown to be prominent not only due to its enhancement in the convergence of fibre backhaul and optical wireless reach extender but also because of its benefit in bi-directional transmission for affording high transmission capacity with long-reach optical wireless link and improved upstream performance.

## Introduction

Broadband access network is considered as an attractive frontier for providing present and emerging technologies, such as fifth-generation mobile communications, immersive virtual reality and augmented reality, 4K video streaming, and high-speed Internet^1–5^. The increasing demands raise the requirement for high transmission capacity, not only for the fibre backhaul but also for the last-mile in-building network. It can be achieved by taking advantages of both optical fibre and optical wireless technologies via the implementation of fibre-free-space optical (FSO) integration^6–8^. In comparison with other systems, fibre-FSO integration can provide not only high transmission capacity but also sufficient flexibility. In this study, a polarization-division-multiplexing (PDM)-based bi-directional fibre-FSO integration with two reflective semiconductor optical amplifiers (RSOAs) scheme to effectually erase the modulated data for uplink transmission is proposed and feasibly demonstrated. For downlink transmission, a high-speed 128 Gb/s vestigial sideband (VSB)-four-level pulse amplitude modulation (PAM4) injection-locked vertical-cavity surface-emitting laser (VCSEL)-based fibre-FSO integration is practically constructed. For an actual realization of fibre-FSO integration, a high-speed operation with improved transmission performance is the critical concern of system operators. The 1.55-μm VCSEL with a single transverse mode has been developed to a few tens of GHz due to its narrow coherent laser beam. Injection locking technique is recognized as one of the most effective techniques for enhancing the modulation bandwidth of VCSEL^9,10^. It integrates the optical properties and advantages of VCSEL and thus opens an access for high-speed operation. As for PAM4 modulation, PAM4 reduces the bandwidth requirement compared with non-return-to-zero (NRZ). Consequently, PAM4 modulation is an efficient approach to meet the high capacity demand. Nevertheless, PAM4 signal possesses a broad linewidth and brings on large fibre dispersion in fibre-FSO integration^11–14^. By contrast, VSB-PAM4 signal possesses a narrow linewidth and brings on small fibre dispersion in fibre-FSO integration^15,16^. The VSB-PAM4 modulation is therefore adopted to mitigate the fibre dispersion impairment, which helps to improve the transmission performance of fibre-FSO integration. Moreover, the polarisation domain has been investigated substantially for fibre-FSO integration. The use of PDM operation is a prominent one to double the channel capacity^17–20^. To inherit the advantages of above-mentioned VSB-PAM4 modulation and PDM operation, the channel capacity of the VSB-PAM4 fibre-FSO integration can be enhanced up to four times.

For uplink transmission, a 10 Gb/s NRZ fibre-FSO integration with two RSOAs scheme for efficiently wiping off the downstream modulated data is practically established. For a real deploying of bi-directional fibre-FSO integration, the receiver side requires an optical carrier for uplink transmission. Wavelength reuse is mostly adopted in bi-directional lightwave transmission systems for upstream. The feasibilities of employing RSOA for wavelength reuse in bi-directional fibre-FSO integration were presented previously^21–23^. However, the upstream performance can be further improved by adopting two RSOAs scheme to effectively erase the modulated data^24,25^. RSOA1 is adopted at the receiver side to erase the downstream data and remodulate the upstream data, whereas RSOA2 is adopted at the transmitter side to erase the modulated data (residual downstream and remodulated upstream data) and reproduce a pure optical carrier to seed into the RSOA1. Two RSOAs scheme enhances the RSOA characteristics and brings on the further erasure of the modulated data in upstream transmission. Given that both downstream and upstream data signals are delivered by the same single-mode fibre (SMF), the Rayleigh backscattering noise limits the upstream transmission performance. Since the further erasure of modulated data, yet the Rayleigh backscattering noise caused by the remodulation reaches the minimum value. Consequently, the upstream performance presents evident improvement. Noticeably, two RSOAs scheme is a promising solution for bi-directional fibre-FSO integration because it can almost erase the modulated data.

A previous study demonstrated a two-way phase-modulated fibre-FSO convergence system with VCSEL-based tunable optical band-pass filter^6^. Another work demonstrated the feasibility of building a bidirectional fibre-wireless and fibre-FSO transmission system with optoelectronic oscillator-based broadband light source and RSOA^22^. Moreover, establishing a bidirectional fibre-wireless and fibre-FSO integrated system with polarization-orthogonal modulation scheme was shown feasibly^26^. Furthermore, a bidirectional fibre-FSO and fibre-wireless convergence system with two orthogonally polarized optical sidebands had been illustrated^7^. However, the downstream data rate/FSO link of 20 Gbps/100 m^6^, 30 Gbps/10 m^22^, 30 Gbps/50 m^26^, and 20 Gbps/100 m^7^ are significantly less than the corresponding values of 128-Gb/s/200-m adopted in this proposed PDM-based bi-directional fibre-FSO integration. In addition, a real-time PAM4 fibre-FSO and fibre-wireless hybrid system with parallel/orthogonally polarized dual-wavelength scheme was presented^27^. Yet, it is difficult for a practical implementation in real networks due to uni-directional transmission. Conclusively, it is necessary to develop a bi-directional fibre-FSO integration with high transmission capacity, sufficient flexibility, and improved upstream performance. A PDM-based bi-directional fibre-FSO integration with two RSOAs scheme for affording high channel capacity (128-Gb/s (downstream)/10-Gb/s (upstream)) with long-reach free-space transmission (200 m) and enhanced upstream performance is thereby demonstrated to meet the targets. For downlink modulation, the channel capacity is enhanced to four times through the PDM operation and VSB-PAM4 modulation. For uplink modulation, the upstream performance shows considerable improvement by using the two RSOAs scheme to erase the modulated data clearly. Bit error rate (BER) (downstream/upstream) and VSB-PAM4 (downstream)/NRZ (upstream) eye diagrams perform well over 25 km SMF transport with 200 m free-space transmission. For in-building network application, a 200-m free-space transmission can greatly satisfy the requirement for in-building transmission distance. Accordingly, this demonstrated PDM-based bi-directional fibre-FSO integration is designed as a promising framework not only due to its enhancement in the integration of fibre backhaul and in-building network but also because of its advantage in two-way communication for providing high transmission capacity with long-range optical wireless link and enhanced upstream performance.

## Results

### Optical power-driving current (P–I) curve and the modulation response of the 1.55-μm VCSEL

**Figure 1:**
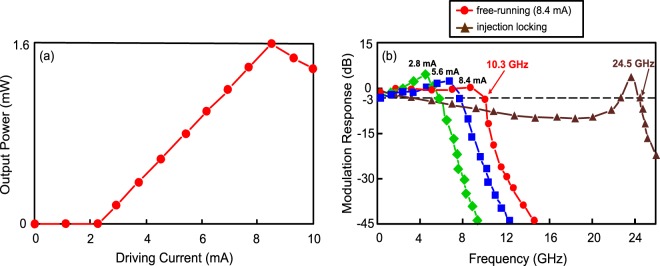
(**a**) P–I curve of the 1.55-μm VCSEL with a threshold current of 2.3 mA and a slope of around 26 mW/mA. (**b**) The modulation responses of the free-running VCSEL under different driving currents and the modulation response of the VCSEL with an injection locking scheme.

Figure 1(a) shows the optical power-driving current (P–I) curve of the 1.55 μm VCSEL with a threshold current of 2.3 mA and a slope of around 26 mW/mA (1.6/(8.4-2.3)). With a driving current of 8.4 mA, a maximum optical output power of 1.6 mW is obtained. Since that the P–I curve is very close to the linear distribution for direct signal modulation, a linear driver with high-linearity is not required to boost the PAM4 electrical signal. Figure 1(b) shows the modulation responses of the free-running VCSEL under different driving currents. It is to be observed that the resonance peak decreases with the increase in driving current. Given that the driving current is 8.4 mA, the 3-dB modulation bandwidth reaches 10.3 GHz. This finding reveals that this 1.55-μm VCSEL is powerful for an effective usage in high-speed fibre-FSO integration. Thus, the driving current of free-running VCSEL is driven at 8.4 mA to acquire a maximum optical output power and a high 3-dB modulation bandwidth. Moreover, the modulation response of the VCSEL with injection locking is presented in Fig. 1(b) as well. Obviously, an injection-locked VCSEL attains a substantial enhancement in 3-dB modulation bandwidth. Under the state of injection locking, a 3-dB modulation bandwidth of 24.5 GHz is achieved. Injection locking technique substantially increases the photon density, by which leading to a considerable 3-dB bandwidth enhancement^28^. The frequency response is featured by a drop in the middle frequencies and a resonance peak in the high frequencies to achieve a considerable 3-dB bandwidth enhancement. Given that the 24.5 GHz is located at the resonance peak, the drop in the middle frequencies will not affect the transmission performances. Since the transmission rate is $\sqrt{2}$ times the bandwidth^29,30^, yet a 128 Gb/s fibre-FSO integration [24.5 ×$\sqrt{2}$× 2 (VSB-PAM4 modulation) × 2 (PDM operation) > 128] can be satisfactorily implemented.

### BER performances and eye diagrams of the PDM-based 128 Gb/s VSB-PAM4 fibre-FSO integration under different scenarios

**Figure 2:**
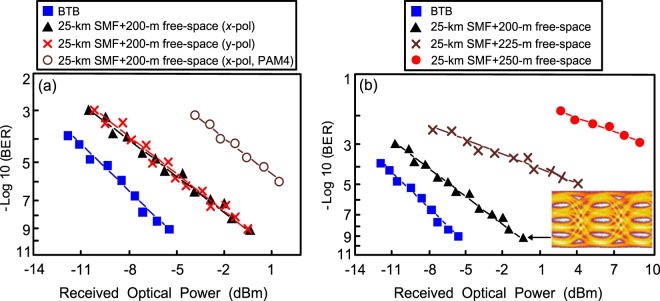
BER performances of the PDM-based 128 Gb/s (**a**) VSB-PAM4/PAM4 fibre-FSO integration for the scenarios of BTB and over 25-km SMF transport with 200-m free-space link (*x*- and *y*- polarisations), and (**b**) VSB-PAM4 VCSEL-based fibre-FSO integration over 25-km SMF transport with different free-space links (*x*-polarisation).

Figure 2(a) presents the BER performances of the PDM-based 128 Gb/s VSB-PAM4/PAM4 fibre-FSO integration for the scenarios of back-to-back (BTB), and over 25-km SMF transport with 200-m free-space link (*x*- and *y*- polarisations). By adjusting the attenuation of variable optical attenuator (VOA) at the transmitter side, different received optical powers can be acquired. The figure shows the BER performances of *x*-polarisation and *y*-polarisation are nearly the same. Therefore, the BER performance’s impact in *x*- and *y*- polarisations is nearly identical. At a BER value of 10^−9^, a power penalty of 5.3 dB exists between the scenarios of BTB and over 25-km SMF transport with 200-m free-space link (*x*- or *y*-polarisation). Such 5.3 dB power penalty results from the use of optical VSB filter to reduce the fibre dispersion by suppressing the linewidth of optical signal. As for signal-to-signal beating interference (SSBI) and cross-beating, no SSBI and cross-beating exist. For two optical sidebands with parallel polarizations, SSBI emerges and degrades the performance of the PDM-based fiber-FSO integration due to the natural feature of two sidebands with parallel polarizations. Given that each polarisation only has one sideband, the SSBI will not emerge. Regarding cross-beating, considering the orthogonality of *x*-polarisation and *y*-polarisation, the cross-beating will be trivially small^27^. Thus, the BER performance will not be degraded by SSBI and cross-beating terms. Analytic result reveals that this proposed PDM-based VSB-PAM4 fibre-FSO integration meets the high accessibility target of fibre backhaul with optical wireless reach extender. Moreover, with PAM4 modulation (*x*-polarisation), the BER value noticeably degrades to 10^−6^. A BER value of 10^−6^ is obtained as a result of the fibre dispersion induced by 25 km SMF transport. Over 25-km SMF transport, distortions caused by fibre dispersion worsens the BER performance because of the natural feature of optical PAM4 signal with broadened linewidth.

The BER performances of the PDM-based 128 Gb/s VSB-PAM4 fibre-FSO integration over 25-km SMF transport with different free-space links (*x*-polarisation) are presented in Fig. 2(b). As the free-space link reaches 200 m, a low BER value of 10^−9^ is acquired. As the free-space transmission link extends to 225 m, the BER degrades to 10^−5^ due to a greater coupling loss at the receiver side, compared to the 200 m free-space link. As the free-space link further extends to 250 m, the BER deteriorates to 10^−3^ because of further coupling losses compared to the 200 m free-space link. The laser light sent from the transmitting side (doublet lens 1) will be spread as it travels a 250-m free-space distance. With spread laser light, the doublet lens 2 collects less light. The received optical power is obviously reduced, by which leading to poor BER performance. Doublet lens, with focal length/back focal length/diameter of 50 mm/50 mm/30 mm, is composed of concave lens and convex lens. Given that the lens has a spatial frequency cutoff (*SFC*) and a corresponding beam radius (*r*), the connection between the *SFC* and the *r* is given by:

1 $$r=2.3\times \frac{1}{SFC\times 2\pi }=3.6\,({\rm{\mu }}{\rm{m}})$$

Moreover, the laser beam divergent angle (*θ*) can be calculated as:

2 $${\theta }=\frac{3.6(\mu m)}{50(mm)}=72\times {10}^{-6}$$

Over a *L*-m FSO link, the laser beam diameter (*d*_*L*_) should be smaller than that of doublet lens 2 (*d*_*L*_ < 30 mm):

3 $$\begin{array}{lllll}{{d}}_{{L}} & = & \sqrt{{{d}}^{2}+{(2{\theta }L)}^{2}} & = & \sqrt{{2.2}^{2}+{(0.144{L})}^{2}} < 30\end{array}$$

where *d* is the laser beam diameter (2.2 mm^31^). *L* is derived as 207.8, representing that the maximum FSO link is 207.8 m. The FSO link adopted in this work is 200 m (<207.8 m) so as to satisfy the maximum FSO link requirement. Through an FSO link, it is critical to match the beam size to the receiving area for avoiding large coupling loss and keeping the free-space link feasibly. If the laser beam size is smaller than the receiving area (FSO link < 207.8 m), then a small coupling loss exists between the laser beam and the ferrule of optical fibre. Therefore, better BER performance is acquired due to more received optical power. By contrast, if the laser beam size is larger than the receiving area (FSO link > 207.8 m), then a large coupling loss exists between the laser beam and the ferrule of optical fibre. Thereby, worse BER performance is acquired due to less received optical power. The eye diagrams of the 64 Gb/s VSB-PAM4 signal over 25-km SMF transport with 200-m free-space link (*x*-polarisation) is displayed in Fig. 2(b) as well. It is to be found that VSB-PAM4 eye diagrams with good quality are observed.

### BER values and eye diagrams of 10 Gb/s NRZ data signal for uplink transmission by varying the RSOA1 seeding power level

**Figure 3:**
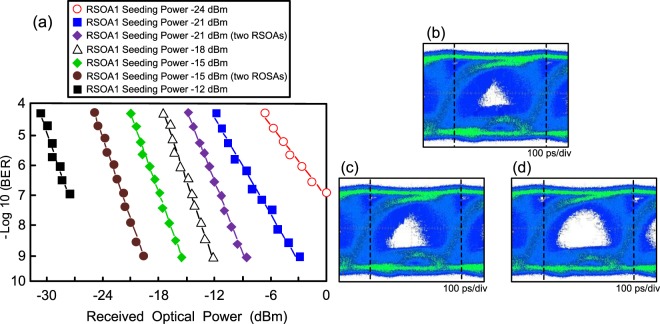
With the adoption of equaliser at the receiver side, for uplink transmission: (**a**) the BER values of 10 Gb/s NRZ data signal by varying the RSOA1 seeding power level from −24 dBm to −12 dBm, and (**b**) the eye diagrams of 10 Gb/s NRZ data signal corresponding to the RSOA1 seeding power level of −24, −15 and −15 (two RSOAs scheme) dBm.

With the adoption of equaliser at the receiver side, the BER values of 10 Gb/s NRZ data signal for uplink transmission by varying the RSOA1 seeding power level from -24 dBm to −12 dBm are shown in Fig. 3(a). To adjust the RSOA1 seeding power level into −21, −18 and −15 dBm, at a BER value of 10^*−*9^, the corresponding received optical power levels are −2.8, −12.1 and −15.4 dBm, respectively. It is to be observed that upstream BER performance improves with the increase in RSOA1 seeding power level. The higher the optical power seeding into the RSOA1, the more suppression the downstream data signal in the upstream transmission attains, thereby improving the upstream BER performance. Whereas if RSOA1 seeding power level is too high, then the RSOA1 will be operated in the gain saturation region. With a seeding power level of −12 dBm, the BER performance can be found to degrade to 10^−7^. A high BER value of 10^−7^ is acquired because of gain saturation-induced nonlinear distortion. In addition, at a BER value of 10^−9^, power penalty improvements of 5.7 dB (RSOA1 with a seeding power level of −21 dBm) and 4.3 dB (RSOA1 with a seeding power level of −15 dBm) exist as two RSOAs scheme is deployed. Two RSOAs scheme brings on further suppression the modulated data in upstream transmission. Consequently, the upstream BER performance is further improved by deploying two RSOAs scheme to wipe off the modulated data effectually.

For uplink transmission, the eye diagrams of 10 Gb/s NRZ data signal corresponding to the RSOA1 seeding power level of −24, −15 and −15 (two RSOAs scheme) dBm, respectively, with the adoption of equaliser at the receiver side are displayed in Fig. 3(b–d). For the state of RSOA1 with a seeding power level of −24 dBm, amplitude and phase fluctuations in eye diagram are obviously observed in Fig. 3(b). Initially, the downstream data signal should be suppressed by RSOA1. Nevertheless, the downstream data signal is ineffectively suppressed because of low optical power seeding the RSOA1, leading to considerable fluctuations in eye diagram. In Fig. 3(c), for the state of RSOA1 with a seeding power level of −15 dBm, amplitude and phase fluctuations are reduced to some extent due to the suppression of the downstream data signal. However, in Fig. 3(d), for the state of RSOA1 with a seeding power level of −15 dBm (two RSOAs scheme), amplitude and phase fluctuations are remarkably reduced due to the effective suppression of the downstream data signal by using two RSOAs scheme.

**Figure 4:**
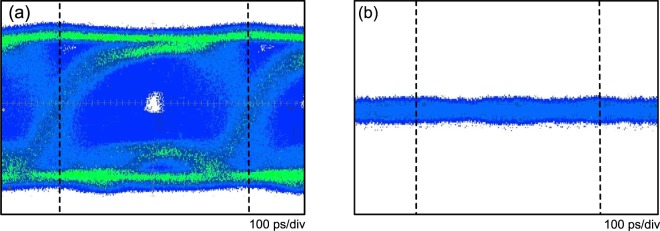
Eye diagrams of 10 Gb/s NRZ data signal measured at the outputs of (**a**) RSOA1 and (**b**) RSOA2 for the state of RSOA1 with a seeding power level of −15 dBm.

To clarify the enhancement achieved using the two RSOAs scheme, a comparison with one RSOA scheme is presented. The eye diagrams of 10 Gb/s NRZ data signal measured at the outputs of RSOA1 and RSOA2 for the state of RSOA1 with a seeding power level of −15 dBm are shown in Fig. 4(a,b). At the output of RSOA1 (Fig. 4(a)), as shown in the eye diagram, large amplitude and phase fluctuations are observed due to the insufficient modulation bandwidth of RSOA1. Since the modulation bandwidth of RSOA1 is only 3.4 GHz, yet an upstream 10 Gb/s fibre-FSO integration [3.4 ×$\sqrt{2}$ < 10] cannot be feasibly realized without the adoption of equaliser at the receiver side. Whereas at the output of RSOA2 (Fig. 4(b)), experimental result shows that the data signal is almost erased, indicating that further erasure of the modulated data in upstream transmission is attained at a data rate of 10 Gb/s.

## Discussion

**Figure 5:**
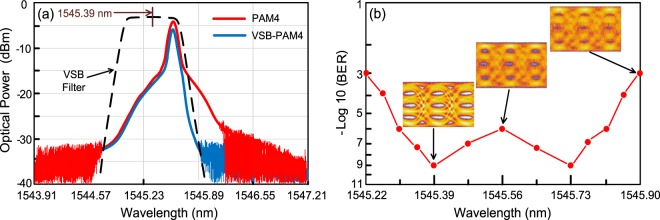
(**a**) Optical spectra of the 64 Gb/s optical PAM4 and VSB-PAM4 signals. (**b**) Measured BER values of the 128 Gb/s fibre-FSO integration over 25-km SMF transport with 200-m free-space link (*x*-polarisation) with different central wavelengths optical VSB filter.

Directly modulated 1.55-μm VCSEL is attractive for the implementation of fibre-FSO integration. However, the transmission performance of the directly modulated VCSEL transmitter-based fibre-FSO integration will be degraded by fibre dispersion-induced distortions due to a long-haul fibre transmission. Therefore, adopting a dispersion compensation device (DCD) within the fibre transmission is necessary to compensate for the fibre chromatic dispersion and reduce the fibre dispersion-induced distortions accordingly^32,33^. Nevertheless, DCD within the fibre transmission will impose the framework complexity on fibre-FSO integration. Developing fibre-FSO integration with simple framework and qualified transmission performance to ensure a successful deployment is required. Optical VSB filter, which can eliminate the redundant linewidth of optical signal, is therefore adopted to effectively reduce the fibre dispersion induced by a 25-km SMF transport. Such proposed optical VSB filter is attractive because it avoids the need of sophisticated DCD within the fibre transmission. The optical spectra of the 64 Gb/s optical PAM4 and VSB-PAM4 signals are displayed in Fig. 5(a). The optical PAM4 signal possesses a broad linewidth, whereas the optical VSB-PAM4 signal possesses a narrow linewidth. The broadened optical spectrum through the interaction of fibre dispersion with the chirp of the directly modulated VCSEL transmitter is compressed by optical VSB filter to enhance the spectral efficiency. Optical VSB filter not only presents high spectral efficiency but also reduces the fibre dispersion by suppressing the linewidth of optical signal. Over 25-km SMF transport, distortions induced by fibre dispersion degrades the transmission performance of fibre-FSO integration due to broadened linewidth of optical PAM4 signal. However, fibre dispersion due to 25 km SMF transport can be mitigated by adopting optical VSB-PAM4 modulation.

To establish a direct connection with the central wavelength of the optical VSB filter and the BER performance, the measured BER values of the 128 Gb/s fibre-FSO integration over 25-km SMF transport with 200-m free-space link (*x*-polarisation) with different central wavelengths of optical VSB filter are displayed in Fig. 5(b). When the central wavelength of the optical VSB filter moves towards 1545.39 or 1545.73 nm, the fibre-FSO integration has the best transmission performance in terms of the lowest BER value and the clearest eye diagrams. As the central wavelength moves towards 1545.39 nm, the optical spectrum of the optical PAM4 signal on the right is partially reduced to form an optical VSB-PAM4 signal. As the central wavelength moves towards 1545.73 nm, the optical spectrum of the optical PAM4 signal on the left is partially reduced to form an optical VSB-PAM4 signal as well. Whereas as the central wavelength moves towards 1545.56 nm, the optical spectrum of the optical PAM4 signal will not be reduced. The optical signal format remains optical PAM4 format. A poor BER performance of 10^−6^ is attained as a result of fibre dispersion caused by 25 km SMF transport. For the state of the central wavelength moving towards a short wavelength (<1545.39 nm) or long wavelength (>1545.73 nm), however, a large part of optical spectrum of the optical PAM4 signal is filtered out. Under such state, the fibre-FSO integration has the worst transmission performance in terms of the highest BER value and the most turbid eye diagrams. A trade-off exists between the linewidth of optical signal and BER performance. Optical signal with narrow linewidth brings on high spectral efficiency but leads to low optical power and high BER value. Optical signal with wide linewidth (PAM4 format) brings on high optical power but results in low spectral efficiency and poor BER performance. Conclusively, optical signal with optimum linewidth (VSB-PAM4 format) brings on optimum optical power and spectral efficiency, and contributes to optimum BER performance. In this work, the central wavelength of the VSB filter is selected at 1545.39 nm so as to gain an optimum trade-off between the linewidth of optical signal and BER performance.

## Methods

### Framework of proposed PDM-based bi-directional fibre-FSO integration with two RSOAs scheme

**Figure 6:**
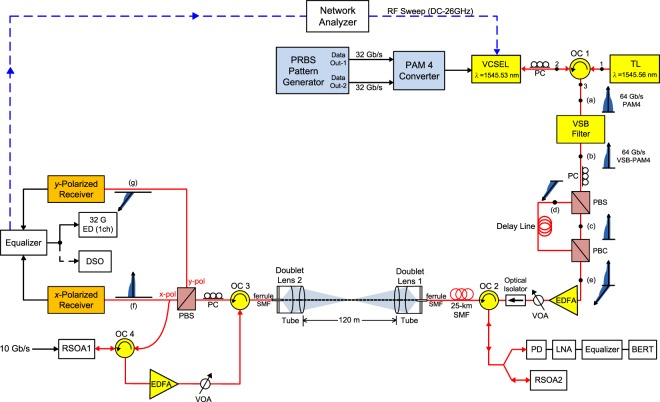
The framework of the proposed PDM-based bi-directional fibre-FSO integration with two RSOAs scheme, and the measurement setup of the frequency response.

The framework of the proposed PDM-based bi-directional fibre-FSO integration with two RSOAs scheme is presented in Fig. 6. Two binary pseudorandom bit sequence (PRBS) data streams with a length of 2^15^*−*1 at 32 Gb/s are generated from a two-channel PRBS generator. These two 32 Gb/s NRZ signals are inputted into a PAM4 converter to generate a 64 Gb/s PAM4 signal. The generated 64 Gb/s PAM4 signal is directly sent to a VCSEL. The light from a tunable laser (master laser, 1545.56 nm) is injected into a VCSEL (slave laser, 1545.53 nm) via a combination of a three-port optical circulator (OC1) and a polarisation controller (PC). The PC is adopted to match the master polarisation to the VCSEL preferred polarisation to verify the stability of injection locking. The optical PAM4 signal [insert (a)] is filtered by a VSB filter to create an optical VSB-PAM4 signal [insert (b)]. The VSB filter is an optical band-pass filter with a 3-dB bandwidth of 0.4 nm and a sharp filter slope of 500 dB/nm. To optimize the optical VSB-PAM4 signal and suppress the fibre dispersion, the central wavelength of the VSB filter is set at 1545.39 nm. In here, the operation of VSB-PAM4 modulation is to enhance the spectrum efficiency and improve the dispersion tolerance. Subsequently, the 64 Gb/s optical VSB-PAM4 signal is separated into two divisions along the two orthogonally polarised sidebands using a PC with a polarisation beam splitter (PBS). The PC is used to adjust the *x*-polarised [insert (c)] and *y*-polarised [insert (d)] sidebands. The *x*-polarised and *y*-polarised sidebands are then recombined by a polarisation beam combiner [insert (e)]. An optical delay line is deployed in the left path to make up for the phase mismatch between two paths. The optical VSB-PAM4 signal with parallel and orthogonally polarised dual sidebands is enhanced by an erbium-doped fibre amplifier (EDFA), controlled by a VOA, guided by an optical isolator, and transported through a 25-km SMF transport with a 200-m free-space transmission via two OCs (OC2 and OC3). The function of the optical isolator is to prevent the optical carrier reflected from the RSOA2. Over a span of 25-km SMF, the optical VSB-PAM4 signal is sent to a 200-m FSO link. The FSO link implements the free-space transmission by using a set of doublet lenses. A set of doublet lenses (doublet lens 1 and doublet lens 2) is utilized to emit laser beam from the ferrule of SMF to the free-space and to conduct laser beam from the free-space to the ferrule of SMF^34^. Thereafter, the 64 Gb/s optical VSB-PAM4 signal is split into two branches along the two orthogonally polarised sidebands utilizing a PC with a PBS. One part of the *x*-polarised sideband with 64 Gb/s optical VSB-PAM4 signal [insert (f)] is received by an *x*-polarised receiver and electrically equalised by a linear equaliser. The operation of linear equalizer is to make amends for the frequency response and increase the transmission rate of VSB-PAM4 fibre-FSO integration^35^. Since that PAM4 linearity is important in the transmission performance, a linear equaliser is adopted to linearize and equalise the electrical VSB-PAM4 signal. After equalisation, real-time BER measurement is executed by using a one-channel error detector (ED). Furthermore, a digital storage oscilloscope is utilized to catch the VSB-PAM4 eye diagrams. Another part of the *x*-polarised sideband is circulated using an OC (OC4), reused and remodulated using a RSOA (RSOA1) for uplink transmission. Similarly, the *y*-polarised sideband with 64 Gb/s optical VSB-PAM4 signal [insert (g)] is received by a *y*-polarised receiver, electrically equalised by a linear equaliser, and detected by a one-channel ED.

For uplink transmission, one part of the *x*-polarised sideband circulated by OC4 is reused and remodulated by RSOA1. A 10 Gb/s NRZ data stream is directly supplied to the RSOA1. Given that the bandwidth of RSOA1 is approximately 3.4 GHz, remodulating 10 Gb/s NRZ data stream using RSOA1 is relatively challenging. With electrical equalisation technique to overcome the bandwidth limitation, however, the RSOA1 can be remodulated at 10 Gb/s NRZ data stream^36,37^. The operation of RSOA1 is to wipe off the downstream modulated data and remodulate the upstream data. The remodulated upstream signal is circulated by OC4, promoted by an EDFA, attenuated by a VOA, circulated by OC3, and launched into a 200-m FSO link with a 25-km SMF transport. Subsequently, the upstream signal is circulated by OC2 and split by a 1 × 2 optical splitter. One part of the remodulated upstream signal with 10 Gb/s NRZ data stream is detected by a 10-GHz photodiode (PD), boosted by a 10-GHz low noise amplifier, equalised by a 10-GHz equaliser, and inputted into a BER tester for BER performance analysis. Another part of the remodulated upstream signal is injected into the RSOA2. The output of RSOA2 is circulated by OC2, blocked by optical isolator, re-circulated by OC2, and seeded into the RSOA1 through 25-km SMF transport with 200-m free-space link. The operation of RSOA2 is to erase the remodulated upstream data and reflect a pure optical carrier back to seed into the RSOA1. Consequently, two RSOAs scheme clearly erase the modulated data to attain remarkably improved upstream performance.

### Measurement setup of the frequency response of PDM-based bi-directional fibre-FSO integration

As to the frequency response, the measurement setup of the frequency response of PDM-based bi-directional fibre-FSO integration with two RSOAs scheme is presented in Fig. 6 as well. RF sweep signal (DC – 26 GHz) generated from a network analyzer is fed into the VCSEL. After electrical equalisation by an equaliser, the RF sweep signal is sent back to the network analyzer. Therefore, the frequency response of the system is measured under the states of free-running VCSEL and injection-locked VCSEL.
